# Breastfeeding Determinants in Healthy Term Newborns

**DOI:** 10.3390/nu10010048

**Published:** 2018-01-05

**Authors:** Lorenzo Colombo, Beatrice Letizia Crippa, Dario Consonni, Maria Enrica Bettinelli, Viola Agosti, Giulia Mangino, Elena Nicoletta Bezze, Paola Agnese Mauri, Lidia Zanotta, Paola Roggero, Laura Plevani, Donatella Bertoli, Maria Lorella Giannì, Fabio Mosca

**Affiliations:** 1 Neonatal Intensive Care Unit, Department of Clinical Sciences and Community Health, Fondazione IRCCS Cà Granda Ospedale Maggiore Policlinico, Università degli Studi di Milano, 20122 Milan, Italy; lorenzo.colombo@mangiagalli.it (L.C.); viola.agosti@hotmail.it (V.A.); giugiu_25_93@hotmail.it (G.M.); elena.bezze@policlinico.mi.it (E.N.B.); lidia.zanotta@policlinico.mi.it (L.Z.); paola.roggero@unimi.it (P.R.); laura.plevani@mangiagalli.it (L.P.); lorella.gianni@mangiagalli.it (M.L.G.); fabio.mosca@mangiagalli.it (F.M.); 2 Epidemiology Unit, Fondazione IRCCS Ca’ Granda Ospedale Maggiore Policlinico, 20122 Milan, Italy; dario.consonni@unimi.it; 3 Mother and Child Unit, Università degli Studi di Milano, ATS Città Metropolitana di Milano, 20122 Milan, Italy; MBettinelli@ats-milano.it; 4 Department of Clinical Sciences and Community Health, Fondazione IRCCS Cà Granda Ospedale Maggiore Policlinico, Università degli Studi di Milano, 20122 Milan, Italy; paola.mauri@unimi.it (P.A.M.); donatella.bertoli@policlinico.mi.it (D.B.)

**Keywords:** breastfeeding, risk factors, protection factors, lactation support, personal experiences

## Abstract

Breastfeeding is the normative standard for infant feeding. Despite its established benefits, different factors can affect breastfeeding rates over time. The purpose of this study was to evaluate breastfeeding determinants in healthy term newborns during the first three months of life. A prospective, observational, single-center study was conducted in the nursery of Fondazione IRCCS Ca’ Granda Ospedale Maggiore Policlinico in Milan, Italy. The mother-baby dyads that were admitted to the Clinic in January and February 2017 were enrolled. Only healthy term babies with birth weight ≥10th percentile for gestational age were included. Data were collected through medical records and questionnaires administered during the follow-up period. Then, we fitted univariate and multivariate logistic models and calculated odds ratios. 746 dyads were included but 640 completed the study. The factors found to be favoring breastfeeding were a previous successful breastfeeding experience, a higher level of education of the mother, attending prenatal classes, no use of pacifier, rooming in practice, and breastfeeding on demand. Factors acting negatively on breastfeeding were advanced maternal age, non-spontaneous delivery, perception of low milk supply, mastitis, and nipple fissures. This study highlights the need to individualize the assistance provide to breastfeeding mothers, paying special attention to personal experiences.

## 1. Introduction

Breast milk is the normal species-specific food for human infants. It is easily absorbed, has a low solute load, and an increased availability of minerals, vitamins, and proteins [1]. Breastfeeding is associated with a reduced risk of child infections, including necrotizing enterocolitis and sudden infant death syndrome [2,3,4,5]; in the long term, a neurodevelopmental advantage and a reduction of the risk of obesity and diabetes in adulthood has been further demonstrated [2,5,6,7,8]. Moreover, breastfeeding provides both short and long-term health benefits to mothers who breastfeed, and it has a positive impact on the society and the ecosystem [2,5,9]. For all of these reasons, the American Academy of Pediatrics reaffirms its recommendation of exclusive breastfeeding for six months after delivery, followed by continued breastfeeding while receiving appropriate and adequate complementary foods [2]. Despite the established benefits of breast milk, global breastfeeding rates remain far below international targets [10], particularly in high-income countries [5], making the identification of modifiable risk factors a high priority [11].

A wide range of historical, socioeconomic, cultural, and individual factors operates at multiple levels and affects breastfeeding decisions and behaviors over time [10]. Women face many barriers to breastfeeding, including lack of public spaces where women can breastfeed without feeling embarrassed; lack of flexible working days for breastfeeding women at work; and, widespread advertising of breast milk substitutes and public policy that ignores the needs of breastfeeding women [10,12]. Great importance has been given to individual factors and personal experiences that can greatly modulate the attitude to breastfeeding [10]. Indeed, awareness of which are the modifiable determinants affecting breastfeeding is essential in managing and supporting the breastfeeding dyad. In this scenario, pediatricians and all health-care providers play a crucial role in their practice as advocates of breastfeeding [2]. The aim of this study was to identify breastfeeding determinants in a population of term, healthy newborns in a tertiary birth center in Milan.

## 2. Materials and Methods 

We conducted a prospective, observational, single-center study in the nursery of Fondazione IRCCS Ca’ Granda Ospedale Maggiore Policlinico in Milan, Italy. The mother-baby dyads admitted to the Clinic in January and February 2017 were enrolled. The study was approved by the Ethics Committee of the Fondazione Istituto di Ricovero e Cura a Carattere Scientifico Cà Granda Ospedale Maggiore Policlinico and written informed consent was obtained from both parents.

We included the healthy babies with gestational age ≥37 weeks, birth weight ≥10th percentile for gestational age, according to the Bertino’s neonatal growth chart [13], and whose mothers were Italian or non-Italian but functionally native Italian speakers, to ensure a full understanding of the questionnaire. Exclusion criteria were the hospitalization in Neonatal Intensive Care Unit and all conditions that could interfere with breastfeeding (including congenital diseases, chromosomal abnormalities, lung disease, brain disease, metabolic disease, cardiac disease, or gastrointestinal diseases).

Data were extracted from obstetric charts and infants’ computerized medical charts (Neocare, I&T Informatica e Tecnologia Srl, Italy). At about 48h after delivery, a structured interview was performed, and a questionnaire was administered: it included closed ended questions allowing mothers to express any difficulties in breastfeeding encountered until then. The variables investigated included sociodemographic features (maternal age, education, ethnicity), previous experiences (participation to a prenatal class and previous experience of breastfeeding), type of delivery, peripartum experiences (rooming in which means keeping the baby in the room for at least 23 h a day and skin-to-skin), and factors affecting lactation (breastfeeding on demand or scheduled breastfeeding, latching difficulties, use of pacifier, nipple fissures, mastitis, and perception of low milk supply). Latching difficulty was defined as the subjective maternal feeling of not being properly latched on. During the hospitalization, this feeling was objectified by a healthcare professional. Gestational age was calculated based on the last menstrual period and recorded. A further follow-up questionnaire was administered at the first visit after discharge. Phone interviews were performed at 15, 40, and 90 days of life. Variables subjected to changes during the timeframe of the study were collected. The mode of breastfeeding was defined according to World Health Organization (WHO) definition as and changes over time were also reported. Five healthcare professionals were involved in the data collection.

The chi-squared test was used to analyze categorical variables. The likelihood of exclusive breastfeeding according to demographic and clinical variables was analyzed with Generalized Estimation Equation (GEE) logistic regression models, to take into account intra-dyad correlation over time. Odds Ratios (OR) with a 95% Confidence Intervals (CI) were calculated with univariate and multiple regression models. All of the statistical analyses were conducted using Stata, version 14 (StataCorp, College Station, TX, USA, 2015).

## 3. Results

The total population included 746 mother-child dyads before discharge (Table 1) but only 640 of them completed the study.

Maternal age ranged from 18 to 49 years. Eighty percent of mothers were Italian and two thirds of them had a degree and attended a prenatal class (66.6% and 66.2%, respectively), while lower frequencies were observed in the population of foreign mothers (35.6% and 44.3%, respectively). Among non-Italian mothers, there was a higher rate of breastfeeding on demand and previous positive experience of breastfeeding when compared to Italian mothers (88.4% vs. 80.4% and 45.9% vs. 28.9%). Moreover, they were more likely to practice rooming in (51.7% vs. 43.7%).

**Figure 1:**
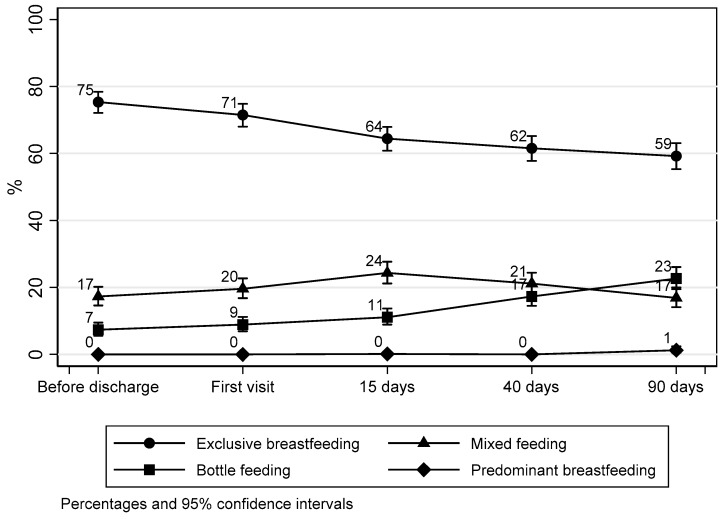
Type of feeding rates during follow-up.

Figure 1 shows the proportion of exclusive breastfeeding reducing over the first three months of life.

Among lactation factors reported in Table 2, incorrect latching of the baby on the breast was more common during the first days after birth, while the perception of low milk supply and mastitis occurring after the first two weeks of life.

Table 3 shows univariate and multivariate regressions. Protective factors for breastfeeding were the following: having a degree or high school diploma, a previous positive experience of breastfeeding, attending prenatal classes, no use of pacifier, rooming in, and a breastfeeding on demand. The increasing of maternal age, a non-spontaneous delivery, the perception of low milk supply, mastitis and nipple fissures represented risk factors for the cessation of breastfeeding.

## 4. Discussion

Awareness of risk and protective factors for breastfeeding is essential for pediatricians and for community and hospital support services. According to the findings of the present study, maternal age, education and prenatal classes attendance, peripartum experiences and delivery, lactation factors, and previous experience of breastfeeding were critical breastfeeding determinants. Our findings expand the present knowledge and contribute to improve support program for mothers.

With regard to maternal age, older mothers are less likely to breastfeed than younger ones in our population. These results are consistent with the literature [14]. However, some studies showed that, in other settings, younger mothers, when compared to older mothers, are at an increased risk of early cessation of exclusive breastfeeding [15,16,17,18,19]. Moreover, disproportionately low breastfeeding rate was reported also among teenage mothers [20] even if this specific category is not well represented in our study. Even though the older age effect on breastfeeding initiation remains to be elucidated [14], it has to be taken into account that an increased maternal age at first childbirth has been recorded in most developed countries in the past 20 years [14], and, hence, a high proportion of mothers aged 35 years or older may require specific attention.

A higher level of education resulted positively associated with breastfeeding. The same trend was observed for prenatal classes attendance. Although no conclusive evidence in literature has been reported supporting any antenatal breastfeeding education for improving initiation of breastfeeding [21], our data suggest starting the promotion of breastfeeding early in pregnancy. Moreover, awareness about the importance of rooming in and skin-to-skin practice could improve women’s chances of successfully breastfeeding, as reported in literature [22].

When considering the factors related to peripartum experiences and delivery, our data indicate a protective effect of practicing rooming in for breastfeeding. However, it has to be taken into account that in the present study, women who did not practice rooming in, represented the majority of the sample, presumably due to the fatigue and the pain that is related to post-partum. Moreover, the number of women who experienced skin-to-skin practice was low. Despite many women in our study experiencing immediate skin to skin contact with their baby, the duration of contact was less than one hour and therefore did not meet the WHO suggested goal for optimal skin to skin contact. Furthermore, the present study confirmed delivery by caesarean section as a risk factor for non-initiating and/or early cessation of breastfeeding [23]. However, as reported by Prior et al. [24], caesarean delivery seems not to affect breastfeeding at six months, suggesting that, in the presence of adequate support, a caesarean delivery is not necessarily a barrier.

Avoiding the use of pacifier in our population represented a protective factor for breastfeeding, and, accordingly, its use should be discouraged. Consistent with these findings, pacifier use has been previously reported to be associated with early weaning, an increase in frequency of ear infections and dental problems [2,25], and “nipple confusion” leading the breastfed infant to struggle with latch on [26]. However, the effect of pacifier exposure on the duration of any breastfeeding is a debated topic. According to WHO recommendations, artificial teats or pacifiers should be totally avoided in breastfeeding infants [27,28]. The American Academy of Pediatrics suggests offering pacifiers to infants at the onset of sleep to reduce the risk of sudden infant death syndrome [29], but recommends avoiding pacifier use until breastfeeding is well established, usually by one month of age [30]. In contrast with our results, pacifier use in healthy term breastfeeding infants, started from birth or after lactation is established, seems not to significantly affect the prevalence or duration of exclusive and partial breastfeeding up to four months of age [31].

The results of our study indicate that breast related problems and the perception of a low milk supply negatively affected breastfeeding, while breastfeeding on demand was strongly associated with the continuation of breastfeeding. The biggest drop in exclusive breastfeeding was observed between the first visit after discharge and 15 days after birth. This decrease may reflect the lack of appropriate support to mothers and suggests that the underlying reason of many of these problems could be incorrect latching of the baby on the breast. In fact, an incorrect latching was more common during the first days after birth, while the perception of reduced milk supply and mastitis occurred after the first two weeks of life. The perception of low milk supply and the feeling that the infant is not satisfied by breast milk alone has been identified as crucial by other authors [32,33,34,35,36] who explore the reasons of an early cessation of breastfeeding. For example, concerns about insufficient milk production were cited by the 21.6% of mothers who stopped breastfeeding completely before six months, as reported by Brown et al. [32]. Similarly, Li et al. reported that when a mother perceives she is not providing an adequate quality or quantity of milk to her infant, she is likely to stop breastfeeding, regardless of infant’s age [33]. The belief that the produced milk is not enough to feed the baby is a further important cause of suspension of breastfeeding in favor of bottle-feeding [32,33,34]. However, <5% of women are biologically incapable of producing a sufficient quantity of milk [37]. The present data indicate that adequate support of breastfeeding, including evaluation of feeding at breast, during the first visit after discharge could prevent the development of fissures and subsequently mastitis and abscesses. Moreover, it is important to give mothers confidence in their own abilities, fully understanding the lactation process, how to latch the baby on the breast properly [38], and how a breastfed baby is able to self-regulate, thus encouraging breastfeeding on demand. What mothers perceive to be a low milk supply may be actually sufficient. In addition, infant growth is uneven and often occurs in spurts [33]. Our results are in line with other studies that highlight the mother’s self-efficacy, which reflects the mother’s confidence in breastfeeding and is closely linked to the personal previous experiences [39,40,41].

A previous positive breastfeeding experience resulted to be a determinant factor for its continuation. A mother who feels confident in breastfeeding will be more likely to repeat the experience and extend the lactation period, with a consequent increase of the associated benefits [10]. Although new mothers frequently experience breastfeeding problems, and many seek support [42], it is interesting to note that a multiparous woman does not necessarily have a high probability of breastfeeding. The relationship between parity or birth order and breastfeeding is not clear [14]. Some studies found that multiparous women were more likely to breastfeed and for longer [18,43]. However, some other studies found no association [44,45]; some reported the opposite association that primiparous mothers were more likely to initiate exclusive breastfeeding at discharge [46], or more likely to continue breastfeeding [47].

In our study, we focused on previous experiences related to parity status. If the previous experience is negative, the mother is less likely to breastfeed than a first-time mother is. Interestingly, it has also been reported that vicarious experience has been shown to influence the behavior of first time and experienced mothers [39]. Therefore, it is important to explore the maternal personal background carefully, identifying any concern arising from previous breastfeeding experiences, and providing tailored support. Although our data were collected from a single institution and it is not possible to generalize our single center findings, a strength of the study is its relatively large number of dyads.

## 5. Conclusions

Our findings reflect a local context of a high-income country where the breastfeeding culture is relatively rooted in the society and its benefits are well known. In this specific social context, we underline the importance of individualizing the assistance provided to mothers, overcoming fixed and rigid rules for a successful breastfeeding and focusing on personal experiences, which can critically affect self-efficacy. In order to confirm the present findings further studies are needed.

## Figures and Tables

**Table 1:** Population features.

Sociodemographic Features	Non-Italian Mothers (*n* = 149)		Italian Mothers (*n* = 597)		Total (*n* = 746)	
	*n*	%	*n*	%	*n*	%
**Maternal age**						
18–29 years	48	32.2	59	9.8	107	14.3
30–34 years	54	36.2	205	34.3	259	34.7
35–39 years	37	24.8	230	38.5	267	35.8
40–49 years	10	6.7	103	17.2	113	15.1
**Education**						
Secondary school diploma	30	20.1	29	4.9	59	7.9
High school diploma	66	44.3	170	28.5	236	31.7
Degree	53	35.6	398	66.6	451	60.4
**Previous experiences**						
**Prenatal classes**						
Yes	66	44.3	395	66.2	461	61.8
No	83	55.7	202	33.8	285	38.2
**Previous breastfeeding experience**						
Positive	67	45.9	172	28.9	239	32.3
None	70	47.9	345	58.1	415	56.1
Negative	9	6.2	77	12.9	86	11.6
Missing data	3		3		6	
**Delivery and peripartum experiences**						
**Type of delivery**						
Spontaneous	79	53	300	50.2	379	50.8
Vacuum/forceps	11	7.4	36	6	47	6.3
Emergency caesarean section	23	15.4	91	15.2	114	15.3
Elective caesarean section	36	24.2	170	28.5	206	27.6
**Gestational age**						
37–38 weeks	58	38.9	274	45.9	332	44.5
≥39 weeks	91	61	323	54.1	414	55.5
**Skin to skin**						
Yes	17	11.4	91	15.2	108	14.5
No	132	88.6	506	84.8	638	85.5
**Rooming in**						
Yes	77	51.7	261	43.7	338	45.3
No	72	48.3	336	56.3	408	54.7
**Lactation factors**						
**Type of breastfeeding**						
Scheduled	17	11.6	116	19.7	133	18
On demand	130	88.4	477	80.4	607	82
Missing data	2		4		6	

**Table 2:** Follow-up findings.

Variable	Before Discharge (*n* = 746)	First Visit after Discharge (*n* = 719)	15 Days of Life (*n* = 711)	40 Days of Life (*n* = 676)	90 Days of Life (*n* = 640)
Lactation factors	*n* (%)	*n* (%)	*n* (%)	*n* (%)	*n* (%)
Latching difficulty	279 (37.4)	117 (16.3)	93 (13.1)	40 (5.9)	18 (2.8)
Pacifier	63 (8.4)	118 (16.4)	178 (25)	266 (39.3)	305 (47.6)
Nipple fissures	53 (7.1)	64 (8.9)	42 (5.9)	19 (2.8)	5 (0.8)
Mastitis	0 (0)	1 (0.1)	16 (2.2)	16 (2.4)	2 (0.3)
Perception of low milk supply	20 (2.7)	34 (4.7)	43 (6)	48 (7.1)	43 (6.7)

**Table 3:** Factors affecting breastfeeding: results of univariate and multivariate logistic regressions.

Sociodemographic Features	OR Crude (95% CI)	OR Adjusted * (95% CI)
**Maternal age**		
Maternal age <30 years	1.0 (reference)	1.0 (reference)
Maternal age 30–34 years	0.7 (0.5–1.1)	0.5 (0.3–0.9)
Maternal age 35–39 years	0.7 (0.5–1.1)	0.5 (0.3–0.8)
Maternal age >40 years	0.5 (0.3–0.8)	0.4 (0.2–0.7)
**Education**		
Secondary school diploma	1.0 (reference)	1.0 (reference)
High School Diploma	1.1 (0.7–1.8)	1.5 (0.8–2.7)
Degree	1.3 (0.8–2)	1.7 (0.9–3.1)
**Ethnicity**		
Non-Italian ethnicity	1.0 (reference)	1.0 (reference)
Italian ethnicity	0.7 (0.5–0.9)	1.0 (0.7–1.5)
**Previous experiences**		
**Prenatal classes**		
No prenatal classes	1.0 (reference)	1.0 (reference)
Prenatal classes	1.5 (1.1–1.9)	1.2 (0.9–1.7)
**Clinical history**		
No breastfeeding experience	1.0 (reference)	1.0 (reference)
Previous negative experience of breastfeeding	0.3 (0.2–0.5)	0.4 (0.2–0.6)
Previous positive experience of breastfeeding	2.4 (1.8–3.3)	2.5 (1.7–3.7)
**Delivery and peripartum experiences**		
**Type of delivery**		
Spontaneous delivery	1.0 (reference)	1.0 (reference)
Vacuum assisted delivery	0.8 (0.5–1.4)	0.9 (0.5–1.6)
Emergency caesarean delivery	0.7 (0.5–1)	0.6 (0.4–0.9)
Elective caesarean delivery	0.5 (0.3–0.6)	0.4 (0.3–0.6)
**Gestational age**		
Gestational age <39 weeks	1.0 (reference)	1.0 (reference)
Gestational age ≥39 weeks	1.1 (0.9–1.5)	0.8 (0.6–1.1)
**Skin to skin**		
No skin to skin	1.0 (reference)	1.0 (reference)
Skin to skin	1.2 (0.9–1.8)	1.0 (0.6–1.7)
**Rooming in**		
No rooming in	1.0 (reference)	1.0 (reference)
Rooming in	1.3 (1.0–1.7)	1.1 (0.8–1.5)
**Lactation factors**		
**Type of breastfeeding**		
Scheduled breastfeeding	1.0 (reference)	1.0 (reference)
On demand	3.7 (2.6–5.2)	3.0 (2.1–4.5)
**Latching**		
No difficulties	1.0 (reference)	1.0 (reference)
Attachment difficulties	1.0 (0.8–1.1)	1.0 (0.8–1.2)
**Pacifier**		
Use of pacifier	1.0 (reference)	1.0 (reference)
No use of pacifiers	1.4 (1.2–1.7)	1.3 (1.1–1.6)
**Nipple fissures**		
No nipple fissures	1.0 (reference)	1.0 (reference)
Nipple fissures	0.9 (0.7–1.2)	0.9 (0.7–1.3)
**Mastitis**		
No mastitis	1.0 (reference)	1.0 (reference)
Mastitis	0.6 (0.4–1.1)	0.6 (0.3–1.1)
**Milk supply**		
No perception of low milk supply	1.0 (reference)	1.0 (reference)
Perception of low milk supply	0.3 (0.2–0.4)	0.3 (0.2–0.4)

* Each factor adjusted for the others. Odds ratios from univariate and multiple Generalized Estimation Equation logistic regression models; OR: Odds ratios; CI: confidence interval.
